# Real World Evidence of Active Surveillance for Prostate Cancer in Spain; Midterm Results

**DOI:** 10.1002/cam4.71173

**Published:** 2025-09-09

**Authors:** J. Rubio‐Briones, A. Borque‐Fernando, L. M. Esteban Escaño, A. Wong, A. Guijarro Cascales, E. Gómez Gómez, J. M. Gil Fabra, F. Sanguedolce, F. Gomez‐Veiga, P. A. López González, A. Plata Bello, N. Rodríguez García, M. Montesino Semper, J. F. Suárez Novo, R. Hajianfar, L. l. Fumadó Ciutat, A. González Alfaro, J. M. Duarte Ojeda, A. Bono Ariño, C. Quicios Dorado, A. Loizaga Iriarte, G. García Fadrique, J. M. Giménez Bachs, S. García Barreras, Y. Pallas Costa, A. Vilaseca Cabo, M. Rodrigo Aliaga, F. Campanario Pérez, P. Servián, J. M. Campá Bortoló, M. Soto Delgado, J. M. Rodríguez de Ledesma, C. Sánchez Rodríguez, V. Chantada Abal, Y. E. Hernández Martínez, B. Herrera Imbroda, P. Dolezal, J. Gual Frau, P. Medrano Llorente, J. Moreno Jiménez, J. S. Serrano Uribe, C. B. Congregado Ruiz, A. Reyes, T. Fernández Aparicio, J. García Rodríguez, M. Cuadras Solé, A. García Seguí, J. J. Pacheco Bru, J. Mayor de Castro, A. Mira Moreno, J. L. Molina Suárez

**Affiliations:** ^1^ Hospital VITHAS 9 de Octubre Valencia Spain; ^2^ Hospital Miguel Servet, IIS‐Aragon Zaragoza Spain; ^3^ Hospital Escuela Universitaria La Almunia Zaragoza Spain; ^4^ Hospital Instituto Valenciano de Oncología Valencia Spain; ^5^ Hospital Fundación Alcorcón Madrid Spain; ^6^ Hospital Reina Sofía Córdoba Spain; ^7^ Hospital Fundación Puigvert Barcelona Spain; ^8^ Hospital Universitario de Salamanca Salamanca Spain; ^9^ Hospital Virgen de la Arraixaca Murcia Spain; ^10^ Hospital Universitario de Canarias Santa Cruz de Tenerife Spain; ^11^ Hospital Son Llatzer Baleares Spain; ^12^ Hospital Virgen del Camino Navarra Spain; ^13^ Hospital Universitario Bellvitge Barcelona Spain; ^14^ Hospital Moises Broggi Barcelona Spain; ^15^ Hospital del Mar Barcelona Spain; ^16^ Hospital Móstoles Madrid Spain; ^17^ Hospital 12 de Octubre Madrid Spain; ^18^ Hospital San Jorge Huesca Spain; ^19^ Hospital Fundación Jiménez Díaz Madrid Spain; ^20^ Hospital Basurto Vizcaya Spain; ^21^ Hospital Manises Valencia Spain; ^22^ Hospital Universitario Albacete Albacete Spain; ^23^ Hospital Ramón y Cajal Madrid Spain; ^24^ Hospital General Universitario Valencia Spain; ^25^ Hospital Clínic Barcelona Spain; ^26^ Hospital General Universitario Castellón Spain; ^27^ Hospital Complejo Asistencial Universitario Palencia Spain; ^28^ Hospital Germans Trias i Pujol Barcelona Spain; ^29^ Hospital Universitario de Araba Alava Spain; ^30^ Hospital Puerta del Mar Cádiz Spain; ^31^ Hospital CST Terrasa Barcelona Spain; ^32^ Hospital Reina Sofía Murcia Spain; ^33^ Hospital CHUAC A Coruña Spain; ^34^ Hospital Manacor Baleares Spain; ^35^ UGC Intercentros Urología Hospitales Universitarios Virgen de la Victoria y Regional Málaga Spain; ^36^ Hospital Barbastro Huesca Spain; ^37^ Hospital Xátiva Valencia Spain; ^38^ Hospital Clínico Lozano Blesa Zaragoza Spain; ^39^ Complejo Hospitalario Jaen Spain; ^40^ Hospital Universitario Príncipe de Asturias Madrid Spain; ^41^ Hospital Universitario Vírgen del Rocío Sevilla Spain; ^42^ Hospital Calatayud, Ernest Lluch Zaragoza Spain; ^43^ Hospital Morales Messeguer Murcia Spain; ^44^ Hospital Universitario Central Asturias Spain; ^45^ Hospital Vall d Hebrón Barcelona Spain; ^46^ Hospital General Universitario Elche Alicante Spain; ^47^ Hospital San Juan Alicante Spain; ^48^ Hospital HGU Gregorio Marañón Madrid Spain; ^49^ Hospital General de Elda Alicante Spain; ^50^ Hospital Vírgen del Puerto Extremadura Spain

## Abstract

**Patients and Methods:**

In this multicenter longitudinal study, data from the Spanish Register in AS (AEU‐PIEM/2014/0001) were reviewed. The study focused on a cohort of AS patients registered between 2014 and 2019, featuring open inclusion criteria and diverse follow‐up strategies.

**Results:**

A total of 3315 AS patients were recruited, with 2881 and 434 categorized into the low and intermediate risk groups based on NCCN grouping at inclusion. The median age was 67 years, and only 11% underwent diagnostic biopsy guided by MRI. The median time between follow‐up visits was 6.03 months. Over a median follow‐up of 62 months (Q1–3: 43.78–85.58), 37% remained in AS, while 8% transitioned to watchful waiting due to aging or intercurrent disease. Death occurred in 199 (6%) of patients, with 3 due to prostate cancer progression and 196 attributed to other causes. At 2 and 5 years, pathological progression‐free survival, metastasis‐free survival, and active treatment‐free survival were 68% and 51%, 99% and 99%, and 70% and 50%, respectively.

**Conclusions:**

Midterm oncological outcomes of AS in Spain align with major international series. We denote underuse of guideline recommendations such as use of MRI or TP Bx for initial PCa characterization. Collaborative efforts are crucial in the search for algorithms, new imaging, or biomarkers to refine indications and transition to active treatments.

**Trial Registration:**

ClinicalTrials.gov identifier: NCT02865330

## 1 Introduction

Many men with early prostate cancer (PCa) show an indolent and long natural history of disease [1]. After 15 years of follow‐up, PCa‐specific mortality is low regardless of the treatment assigned [2]. In the 90's, active surveillance (AS) strategies started to gain popularity regardless of age in the Urologic community, differentiating it from just watchful waiting (WW) policies. Its rationale and clear objective were to reduce overtreatment, side effects of active management, and costs [3].

As no single protocol for AS has been shown to be superior, allowing for variations in practice, the Spanish Register on AS (AEU‐PIEM/2014/0001 [4]) acts as a real world evidence (RWE) Register with wide inclusion criteria, different follow‐up (FU) strategies, and criteria for active treatment. It includes patients with low and intermediate‐risk PCa and assumes disease progression as tumor size increases or as a worsening in the biopsy score (upgrading of the Grade Group) [5].

There is a clear need to incorporate RWE data in AS in every country with different public health systems and policies. For example, following classic AS protocols, its costs were roughly half of the 5‐year costs of radical prostatectomy in Spain. But this classic concept of the more cost‐effective behavior of the AS strategy has been recently questioned at 5‐year FU data of AS in real world evidence (RWE), due to increasing costs of magnetic resonance imaging (MRI) and transperineal fusion biopsies based FU protocols [6].

The main present study aim is acknowledging midterm data within our Register in terms of different survival endpoints investigating real world data in our country. This will give urologists RWE data for counseling AS, will provide figures to calculate resources and cost for Health authorities, and finally will find room for improvement in front of low and intermediate newly diagnosed PCa.

## 2 Material and Methods

### 2.1 Patient and Data Recruitment

Briefly, as previously published [4], the Spanish Register in AS started recruitment in July 15, 2014, admitting patients included in AS protocols retroactively and prospectively. It closed recruitment 5 years later, on July 15, 2019. Collection of data for this report was done in June 23, 2022. Patients for any of the 48 participating centers all along the country were included if PSA was ≤ 20 ng/mL, digital rectal examination (DRE) dictated clinical stage (cT) of cT1a‐b‐c or cT2 a‐b‐c, and primary Gleason pattern was 3 and secondary 3 or 4. Prostate volume was calculated from hypogastric or transrectal ultrasound. Initial magnetic resonance imaging (MRI) was not mandatory as per the studied period but was registered in follow‐up biopsies when used. A minimum of 10 diagnostic cores were mandatory to be included, and patients could harbor 1, 2, or 3 positive cores. No central pathological review was considered. Follow‐up strategies were open with no pre‐determined timing for follow‐up biopsies (Bx) nor type of Bx approach, mirroring real‐life practices in our country. Transition to watchful waiting strategy relied on the investigator's judgment due to intercurrent disease or at the age of 80. Patients who only underwent the diagnostic visit were considered lost to follow‐up.

Pathological progression was defined as any increase in Gleason Grade Group (GG) or within the same GG if any core had a PCa length > 5 mm or 50% in cores at least 1 cm in length, 3 or more affected cores in any FU transrectal ultrasound guided Bx (TRUS‐Bx) or 3 or more affected cores and more than 2 affected prostatic zones if FU Bx had a transperineal approach (TP Bx). Local progression was defined as increase by FU DRE cT or MRI from cT1‐2 to cT3. To define metastatic progression, metastasis had to be diagnosed by bone scan, computed tomography (CT) or MRI scan or next generation imaging techniques such as positron emission tomography (PET) with choline or PSMA as radiotracers.

### 2.2 Statistical Analysis

Since this study is observational in nature, the analysis primarily adopts a descriptive approach. Characteristics of the whole cohort and by risk categories were described using median and interquartile range (percentile 25th‐percentile 75th) and absolute and relative frequencies, for continuous and categorical variables respectively. Differences by low and intermediate risk groups were analyzed using Mann–Whitney or Fisher exact test as appropriate. Registered data sometimes are missing due to open label and not central research office guided implementation; thus, when we refer to percentages we take in account reliable registered data.

The period time between biopsies were visualized using violin plots and the results of the follow‐up biopsies were described using bar plots. The patient status at the time of analysis were analyzed by pie chart.

The pathological, local progression, active treatment, metastasis, overall mortality, and cancer specific free survival probabilities were calculated and displayed using Kaplan–Meier survival curves.

Data analysis were carried out using version 4.0.3 of the R statistical software, through version 1.3.1093 of the RStudio development environment (RStudio Team (2021). RStudio: Integrated Development for R. RStudio Inc., Boston, MA, USA, URL http://www.rstudio.com/).

The study was reviewed and approved by the institutional review board of Ethics Committee for Clinical Research of Aragon (Reg. No. PI17/0280), registering with the code AEU‐PIEM/2014/0001.

## 3 Results

### 3.1 Diagnosis

During the 5‐year recruitment period, a total of 3315 patients were included in the Register in 48 Centers. The median age of the participants was 67 years (P25‐75, 61–71), with 99.6% being Caucasian. The majority (83.92%) resided in urban areas, while only 17% had university‐level education. Most patients had a stable partner (93%), and 43% engaged in sporadic or regular sports activities. Out of the patients, 343 (10%) were lost of FU. When asked about familial prostate cancer, 14% acknowledged having relatives with the condition. Table 1 presents the main clinico‐pathological variables at inclusion. According to the National Comprehensive Cancer Network (NCCN) prognostic groups, 434 patients (13.09%) belonged to the intermediate‐risk group, while 2281 patients (89.91%) fell into the very low and low‐risk groups. At recruitment, the median PSA level was 6.05 (IQR: 4.75–7.94) ng/mL. The most common cT stage was cT1c (88%), while 265 patients (8%) had cT2 stage. The majority of patients were minimally or mildly symptomatic, with a mean IPSS score of 9.3 (SD 6.31) and a mean quality of life score of 1.86 (SD 1.38). Approximately 41% of patients were receiving medical treatment for lower urinary tract symptoms (LUTS), while only 7% had undergone surgery for LUTS. For the initial diagnosis, transrectal ultrasound‐guided biopsy (TRUS‐Bx) was used in 97% of men, while transperineal biopsy (TP‐Bx) was used in only 3% of cases. Among the available data, only 371 patients (11%) had their diagnostic biopsy guided by MRI. Among these patients, PI‐RADs 3–5 lesions were observed in 194 individuals (52%). The use of MRI increased to 1207 patients (36%) with FU for the confirmatory biopsy. At diagnosis, the most frequent Gleason Grade group was 1 (3 + 3), observed in up to 97% of the series.

**TABLE 1:** characteristics of the series at diagnosis.

	Whole series	Low risk	Intermediate risk
N	3315	2881 (86.91%)	434 (13.09%)
Age (years)	Median 67 Q1–Q3; 61–71 Min/Max; 36/86	Median 66 Q1–Q3; 61–71 Min/Max; 36/86	Median 69 Q1–Q3; 63–72 Min/Max; 41/84
ECOG	0; 1726 (52.07%) I; 209 (6.30%) II; 18 (0.54%) III; 2 (0.06%) N.A; 1360 (41.03%)	0; 1511 (52.45%) I; 182 (6.32%) II; 17 (0.59%) III; 1 (0.03%) N.A; 1170 (40.61%)	0; 215 (49.54%) I; 27 (6.22%) II; 1 (0.23%) III; 1 (0.23%) N.A; 190 (43.78%)
BMI	Median 27.34 Q1–Q3; 25.01–29.75 Min/Max; 15.81/50.92 N.A; 1579 (46.73%)	Median 27.38 Q1–Q3; 25.01–29.76 Min/Max; 15.81/50.92 N.A; 1341 (40.45%)	Median 26.93 Q1–Q3; 25.00–29.62 Min/Max; 20.90/40.27 N.A; 208 (47.93%)
PSA (ng/ml)	Median 6.05 Q1–Q3; 4.75–7.94 Min/Max; 0.50/20.50 N.A; 0 (0%)	Median 5.80 Q1–Q3; 4.62–7.25 Min/Max; 0.50/10.00 N.A; 0 (0%)	Median 11.40 Q1–Q3; 10.07–13.64Min/Max; 0.85/20.50 N.A; 0 (0%)
cT	cT1a; 77 (2.32%) cT1b; 34 (1.03%) cT1c; 2912 (87.84%) cT2a; 234 (7.06%) cT2b; 16 (0.48%) cT2c; 15 (0.45%) N.A; 27 (0.81%)	cT1a; 70 (2.43%) cT1b; 31 (1.08%) cT1c; 2552 (88.58%) cT2a; 207 (7.19%) cT2b; 0 (0%) cT2c; 0 (0%) N.A; 21 (0.73%)	cT1a; 7 (1.61%) cT1b; 3 (0.69%) cT1c; 360 (82.95%) cT2a; 27 (6.22%) cT2b; 16 (3.69%) cT2c; 15 (3.46%) N.A; 6 (1.38%)
Prostate volume (cc)	Median 45 Q1–Q3; 33–62 Min/Max; 9/250 N.A; 0 (0%)	Median 45 Q1–Q3; 33–60 Min/Max; 9/250 N.A; 0 (0%)	Median 51 Q1–Q3; 37–80 Min/Max; 13/200 N.A; 0 (0%)
PSA density	Median 0.13 Q1–Q3; 0.09–0.19 Min/Max; 0.01/1.09 N.A; 0 (0%)	Median 0.13 Q1–Q3; 0.09–0.18 Min/Max; 0.01/0.09 N.A; 0 (0%)	Median 0.19 Q1–Q3; 0.13–0.29 Min/Max; 0.02/1.09 N.A; 0 (0%)
Positive cores (%)	Median 8.33 Q1–Q3; 8.00–15.79 N.A; 12 (0.36%)	Median 8.33 Q1–Q3; 8.33–16.67 N.A; 10 (0.35%)	Median 8.33 Q1–Q3; 6.00–11.11 N.A; 2 (0.46%)
GG 1 GG 2	Grade 6; 3210 (96.83%) Grade 7; 96 (2.90%) N.A; 9 (0.27%)	Grade 6; 2872 (99.69%) Grade 7; 0 (0%) N.A; 9 (0.31%)	Grade 6; 338 (77.88%) Grade 7; 96 (22.12%) N.A; 0 (0%)
PCa length/core (mm)	Median; 1.6 Q1–Q3; 1–3 N.A; 1619 (48.84%)	Median; 1.7 Q1–Q3; 1–3 N.A; 1358 (40.97%)	Median; 1.2 Q1–Q3; 1–3 N.A; 261 (60.14%)
Percentage PCa/core	Median; 10 Q1–Q3; 5–20 N.A; 1403 (42.32%)	Median; 10 Q1–Q3; 5–20 N.A; 1205 (41.83%)	Median; 8.34 Q1–Q3; 5–20 N.A; 198 (45.62%)

Abbreviations: BMI, body mass index; cT, clinical stage; GG, Gleason Grade Group; N.A, not available; PSA, prostatic specific antigen; Q, quartile.

### 3.2 Follow‐Up

**FIGURE 1:**
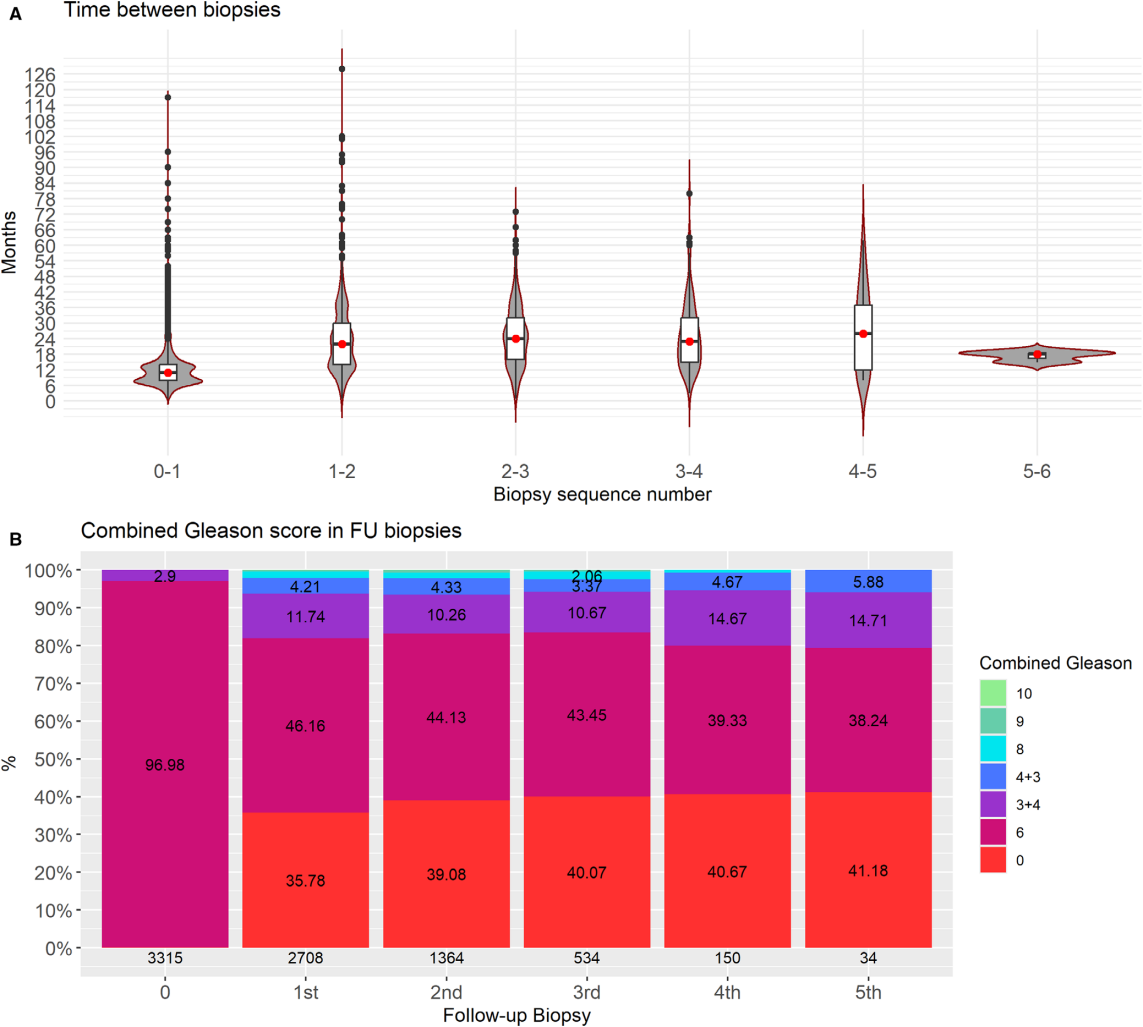
(A) Time between biopsies; (B) combined Gleason score in follow‐up biopsies.

We had a median FU of 62.13 months (Q1–3: 43.78–85.58), Median time elapsed between FU visits was 6 months (Q1–3; 4.13–7.93). This time frame between FU visits was stable along most FU in different Centers. In Figure 1A we display time elapsed between FU Bx, observing that the first (confirmatory) Bx had a median of 11 months (IQR: 8–14) and the median between the following was around 22 months. Taking in account those 2708 patients with FU Bx, 1344 (50%) had just one, and then 830 (31%), 384 (14%), 116 (4%) and 34 (1%) had 2, 3, 4 and 5 or more respectively. During the first year of FU, 1424 men with FU were biopsied (47%), progressively reducing the percentage of biopsied men in the following years. When focusing on type of Bx in FU, TP‐Bx approach was more frequently used (17%), but still far from TRUS. In Figure 1B, we can see pathological results of the FU Bx; the percentage of non PCa results was 36% in first FU Bx, slowly increasing to 41% in fifth FU Bx. The percentage of GG 2 or higher was stable around 15% in the three first FU Bx, but increasing between posteriorly up to 21% at the 5th.

**FIGURE 2:**
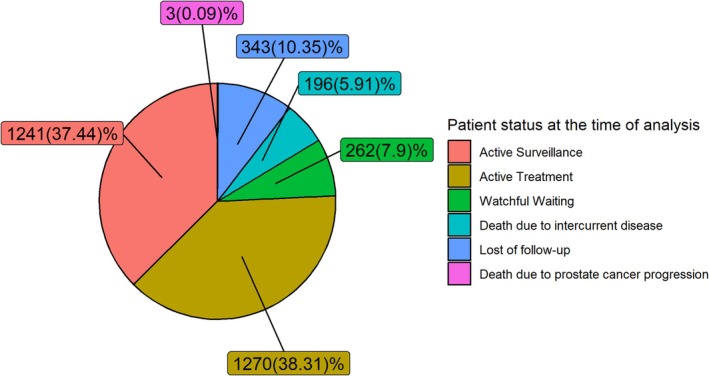
Patient status at the time of analysis.

In Figure 2 we can see final stage of the whole. With a median FU of 62 months (Q1–3: 43.78–85.58), we observed that just 1241 patients (37.44%) remain in AS, while 1270 have been actively treated (38.31%). Two hundred sixty‐two (7.69%) have been transferred to a watchful waiting (WW) strategy due to aging or intercurrent disease. Death occurred in 199 patients (6%), 3 per PCa progression and 196 for other causes.

### 3.3 Progression Analyisis

**FIGURE 3:**
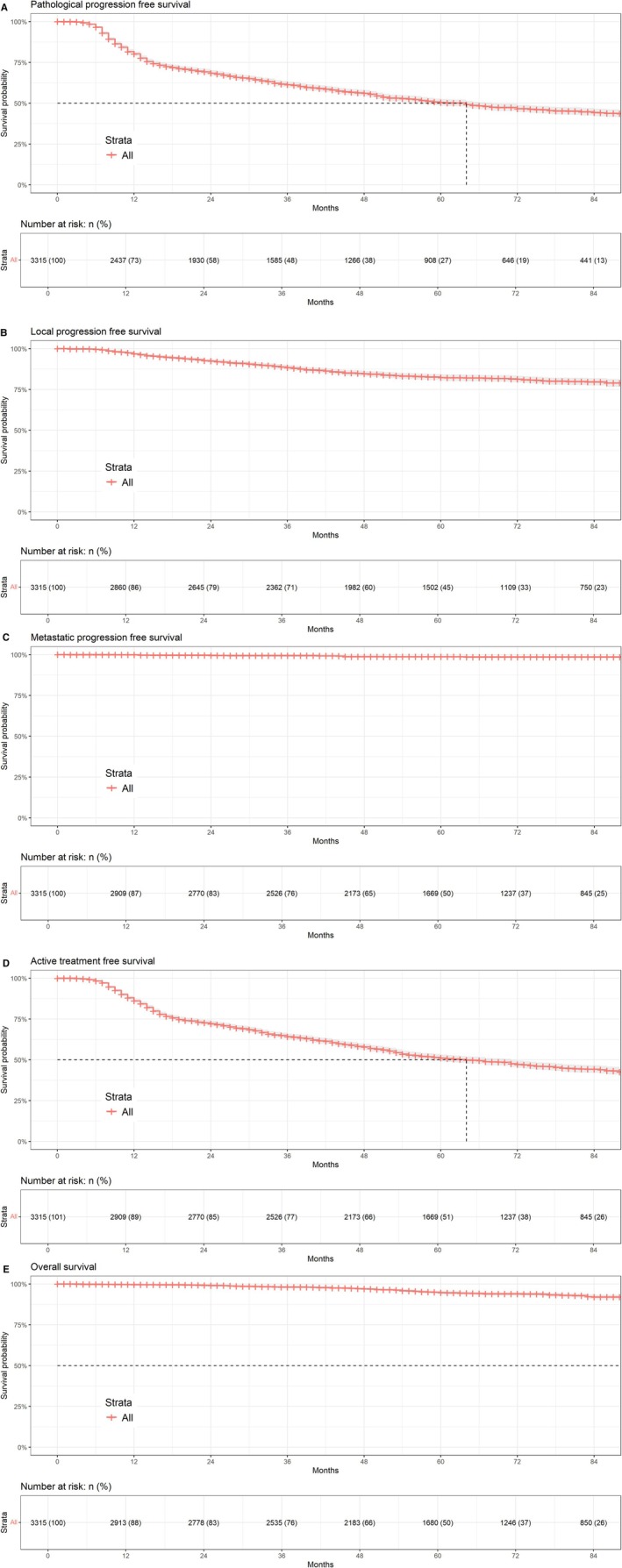
(A) Pathological progression free survival. (B) Local progression free survival. (C) Metastatic progression free survival. (D) Active treatment progression free survival. (E) Overall survival.

Pathological progression occurred in 1450 (48.5%) out of 2998 available FU data patients, resulting in a pathological progression free survival at 2 and 5 years of 68% and 51% respectively (Figure 3A). Local progression occurred in 371 patients (12.4%), and local progression free survival at 2 and 5 years were 94% and 88% (Figure 3B). Metastasis occurred in 21 patients out of 2998 (0.7%), with corresponding metastasis free survival rates at 2 and 5 years of 99% and 99% respectively (Figure 3C). Active treatment free survival at 2 and 5 years were 71% and 50% respectively, as 1426 were treated somehow (47.6%) (Figure 3D). Just 3 patients died from PCa. Finally, overall survival at 2 and 5 years were 99% and 95% respectively (Figure 3E).

Among the 1426 active treatments performed, radical prostatectomy was the most frequent used in 647 patients (45%), followed by radiotherapy (419, 29%), brachytherapy (101, 7%), hormonotherapy (31, 2%), focal treatment (29, 2%) and cryotherapy (8, 0.56%) (Figure S1A); in Figure S1B, we show second active rescue treatments in 50 patients after progression from the first active treatment in row numbers and percentages.

Out of the 1270 patients receiving active treatment with available data, 202 (15.9%) exhibited no pathological progression. On the other hand, within the group of patients with pathological progression, 27.2% experienced progression without receiving active treatment, while within the group of patients with local progression, 36.3% showed progression without active treatment.

## 4 Discussion

In 2022, the better understanding of the highly varied natural behavior [7] of low risk PCa and the morbidities associated with PCa treatments [8] has led to increasing embracement of AS protocols. So AS is now considered the preferred management strategy for low‐grade (i.e., GG 1) PCa. But that was not so clear when our Registry was planned in 2012 to 2013, a time frame where AS data started to be considered in major Guidelines. It prompted urologists in our country to take AS into consideration as one of the first options, if not mandatory, in very low and low risk PCa patients. As mentioned, its main aim was to promote AS use and attending to its wide implementation today, it was successfully accomplished mirroring what has happened in many developed countries [9].

The previous was the reason to allow as much Centers as possible, avoiding closed and strict FU protocols that just had hampered recruitment and, consequently, AS promotion. The concept of promoting AS for low‐risk and favorable intermediate‐risk PCa patients is still a relevant issue; just to remember, AS embracement is poor in underdeveloped countries and, in 2022, nearly half of men on active surveillance for favorable‐risk prostate cancer do not receive all recommended surveillance [10] tools. Another worrisome concept we glimpse is the current debate to remove the label of “cancer” from Gleason 3 + 3 lesions [11]. We do agree with arguments against this from the editors of the 2022 edition of the WHO classification of urinary and male genital tumors [12]. We think that just following recent EU recommendations for PCa screening [13], retaining the term cancer assigned to GS 6/GG1 prostate adenocarcinoma and providing subsequent organized and individualized AS programmes to tamponade overtreatment, low‐ risk and intermediate‐risk PCa management will reproduce solid known figures on prostate cancer mortality and even improve them, facing, as we all know, an encouraging progressive increase in men's life expectancy in developed countries [14].

We find interesting our RWE data showing that AS oncological figures in Spain mirror major AS in the literature, considering a non‐organized protocol supporting this fact. With just 21 and 3 out of 3315 patients having had metastasis and died due to PCa [15, 16, 17, 18, 19].

We show the most relevant and general figures describing the diagnosis and follow‐up of patients, trying to find out where management of AS might be improved. Recognizing that a median FU of 62 months (Q1–3: 43.78–85.58) is not enough for PCa specific mortality (PCSM), we would like to observe a perfect overlapping between pathological progression and active treatment‐free survival curves (Figure 3A,D). We performed 4794 FU biopsies and just 746 (15.56%) had a combined Gleason score of 7. Attending to the results of a recent meta‐analysis, highlighting the role of GG 3 or higher as the “real fail” in an AS programme [20] due to its relationship with worse oncological behavior, we find our active treatment rates somehow worrisome, as we had just 202 (4.21%) GG 3 in FU Bx when we split combined Gleason 7 in our FU Bx into 3 + 4 versus 4 + 3. We and others think that progression in GG1 volume or an isolated non‐massive upgrading to GG2 has to be deeply discussed with the patient before moving to active treatment, considering other recognized factors of bad behavior such as MRI visibility [21] and PSA‐density [22].

Acknowledging the debate of intermediate risk definition [23], our 13% of patients recruited as the intermediate risk group mirrors what occurs in other collaborative groups. We are aware that pathological progression to GG2 should be further discussed with the patient, as many of them are still not clinically significant. Maybe we should keep in mind that when managed with watchful waiting, intermediate‐risk PCa is associated with 10‐year and 15‐year PCSM rates of 13% and 20% [24, 25], so probably age at progression is a matter. Huge efforts are being displayed nowadays in what we understand as a “second wave” AS era pursuing new biomarkers or algorithms that could split aggressive from indolent PCa.

The main strength of our Registry is the representation of RWE data in our country, assuming many limitations inherent to its nature and design. Our shared results allow RWE data for urologists to inform new candidates for AS today. MRI use is similar to our series in many AS published reviews [20]. Although the use of MRI and TP Bx increased in our FU Bx, these tools still are not available in every Center in Spain, so our data confer reliable information to clinicians. Future research will evaluate the results of AS patients by incorporating the latest tools such as MRI, better characterization of family history of PCa, and genomic testing [26], or new biomarkers. It has recently been pointed out that the inclusion of MRI in the diagnostic PCa‐care pathway is likely to cause risk inflation, compromising AS enrollment [27, 28]. We think RWE data has to promote artificial intelligence‐based tools to personalize AS biopsy schedules for each AS candidate, avoiding decisions based just on MRI data, which is current nowadays. These tools might optimize AS protocols, also increasing costs [6], but if their use does improve oncological figures of classic AS protocols without them, still remains to be proven.

## 5 Conclusions

Midterm RWE oncological results of AS in Spain do mirror those in major series worldwide following a non‐standardized protocol. We denote underuse of Guideline recommendations such as use of MRI or TP Bx for initial PCa characterization. We prompt collaborative work searching for algorithms, new imaging, or new biomarkers that could shed some light to avoid unnecessary active treatment during surveillance.

## Author Contributions

**José Rubio‐Briones:** conceptualization (lead), data curation (lead), formal analysis (lead), funding acquisition (lead), investigation (lead), methodology (lead), project administration (lead), resources (lead), supervision (lead), writing – original draft (lead), writing – review and editing (lead). **Angel Borque‐Fernando:** conceptualization (lead), data curation (lead), formal analysis (lead), funding acquisition (lead), investigation (lead), methodology (lead), project administration (lead), resources (lead), supervision (lead), writing – original draft (lead), writing – review and editing (lead). **Luis Mariano Esteban‐Escaño:** conceptualization (lead), data curation (lead), formal analysis (lead), investigation (lead), methodology (lead), writing – review and editing (equal). **A. Wong:** investigation (equal), writing – review and editing (equal). **A. Guijarro Cascales:** investigation (equal), writing – review and editing (equal). **E. Gómez Gómez:** investigation (equal), writing – review and editing (equal). **J. M. Gil Fabra:** investigation (equal), writing – review and editing (equal). **F. Sanguedolce:** investigation (equal), writing – review and editing (equal). **F. Gomez‐Veiga:** investigation (equal), writing – review and editing (equal). **P. A. López González:** investigation (equal), writing – review and editing (equal). **A. Plata Bello:** investigation (equal), writing – review and editing (equal). **N. Rodríguez García:** investigation (equal), writing – review and editing (equal). **M. Montesino Semper:** investigation (equal), writing – review and editing (equal). **Jose Francisco Suárez:** investigation (equal), writing – review and editing (equal). **R. Hajianfar:** investigation (equal), writing – review and editing (equal). **L. l. Fumadó Ciutat:** investigation (equal), writing – review and editing (equal). **A. González Alfaro:** investigation (equal), writing – review and editing (equal). **Jose Manuel Duarte‐Ojeda:** investigation (equal), writing – review and editing (equal). **A. Bono Ariño:** investigation (equal), writing – review and editing (equal). **C. Quicios Dorado:** investigation (equal), writing – review and editing (equal). **A. Loizaga Iriarte:** investigation (equal), writing – review and editing (equal). **G. García Fadrique:** investigation (equal), writing – review and editing (equal). **Jose Miguel Gimenez‐Bachs:** investigation (equal), writing – review and editing (equal). **Silvia García Barreras:** investigation (equal), writing – review and editing (equal). **Y. Pallas Costa:** investigation (equal), writing – review and editing (equal). **Antoni Vilaseca:** investigation (equal), writing – review and editing (equal). **M. Rodrigo Aliaga:** investigation (equal), writing – review and editing (equal). **F. Campanario Pérez:** investigation (equal), writing – review and editing (equal). **P. Servián:** investigation (equal), writing – review and editing (equal). **J. M. Campá Bortoló:** investigation (equal), writing – review and editing (equal). **M. Soto Delgado:** investigation (equal), writing – review and editing (equal). **J. M. Rodríguez de Ledesma:** investigation (equal), writing – review and editing (equal). **C. Sánchez Rodríguez:** investigation (equal), writing – review and editing (equal). **V. Chantada Abal:** investigation (equal), writing – review and editing (equal). **Y. E. Hernández Martínez:** investigation (equal), writing – review and editing (equal). **Bernardo Herrera‐Imbroda:** investigation (equal), writing – review and editing (equal). **P. Dolezal:** investigation (equal), writing – review and editing (equal). **J. Gual Frau:** investigation (equal), writing – review and editing (equal). **P. Medrano Llorente:** investigation (equal), writing – review and editing (equal). **J. Moreno Jiménez:** investigation (equal), writing – review and editing (equal). **J. S. Serrano Uribe:** investigation (equal), writing – review and editing (equal). **C. B. Congregado Ruiz:** investigation (equal), writing – review and editing (equal). **A. Reyes:** investigation (equal), writing – review and editing (equal). **T. Fernández Aparicio:** investigation (equal), writing – review and editing (equal). **J. García Rodríguez:** investigation (equal), writing – review and editing (equal). **M. Cuadras Solé:** investigation (equal), writing – review and editing (equal). **A. García Seguí:** investigation (equal), writing – review and editing (equal). **J. J. Pacheco Bru:** investigation (equal), writing – review and editing (equal). **J. Mayor de Castro:** investigation (equal), writing – review and editing (equal). **A. Mira Moreno:** investigation (equal), writing – review and editing (equal). **J. L. Molina Suárez:** investigation (equal), writing – review and editing (equal).

## Conflicts of Interest

The authors declare no conflicts of interest.

## Supporting information


**Figure 1A.** First active treatment received.

**Figure 1B.** Second active treatment recieved.

## Data Availability

The data that support the findings of this study are available from the corresponding author upon reasonable request.
