# Supplementary figures and images for: Real World Evidence of Active Surveillance for Prostate Cancer in Spain; Midterm Results
Source: Cancer Med. 2025 Sep 9;14(17):e71173. doi: 10.1002/cam4.71173 (PMC12420667; doi:10.1002/cam4.71173)

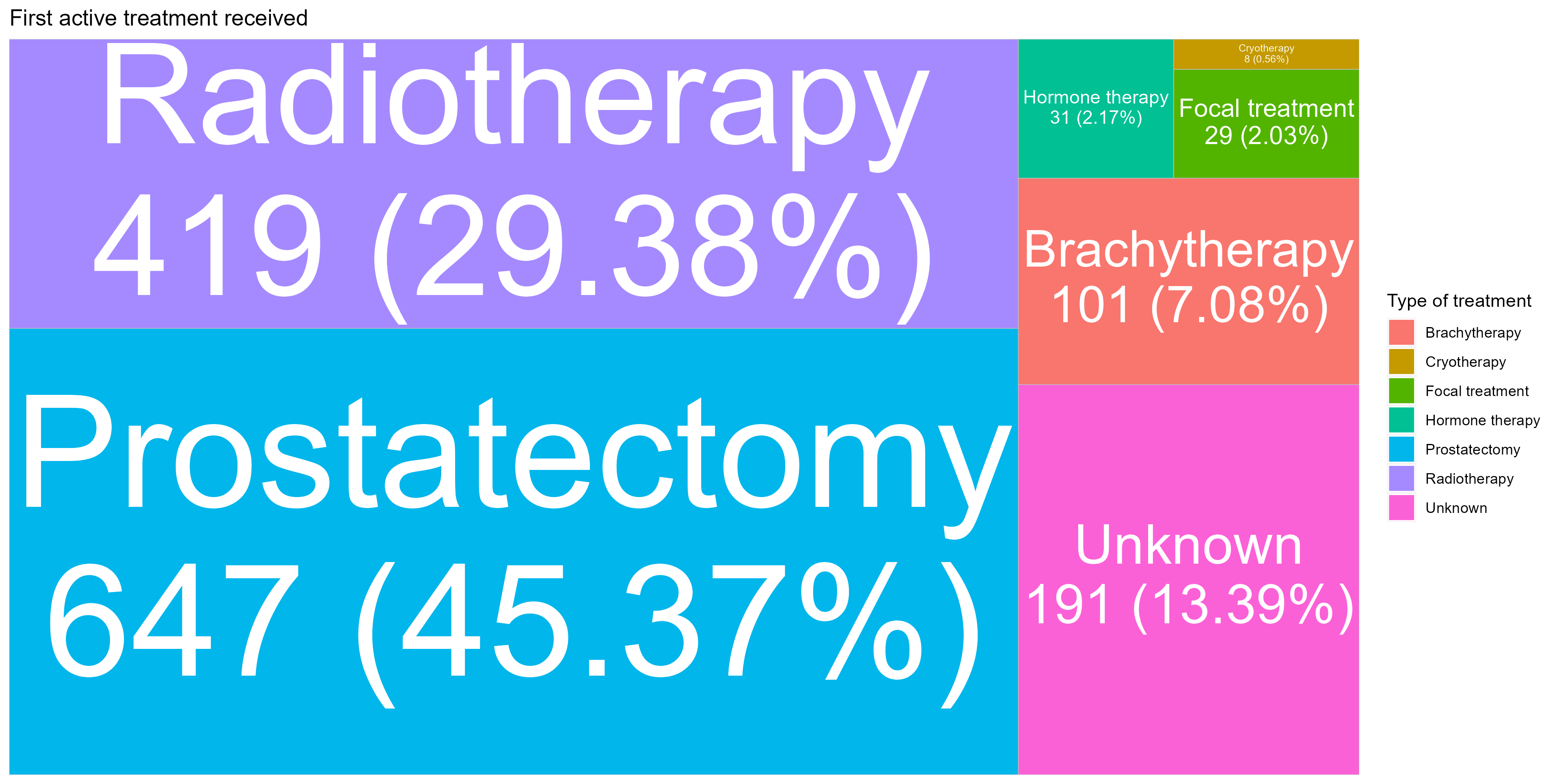

Supplement: Supplementary file 1 — Figure 1A. First active treatment received. [file CAM4-14-e71173-s001.tiff]

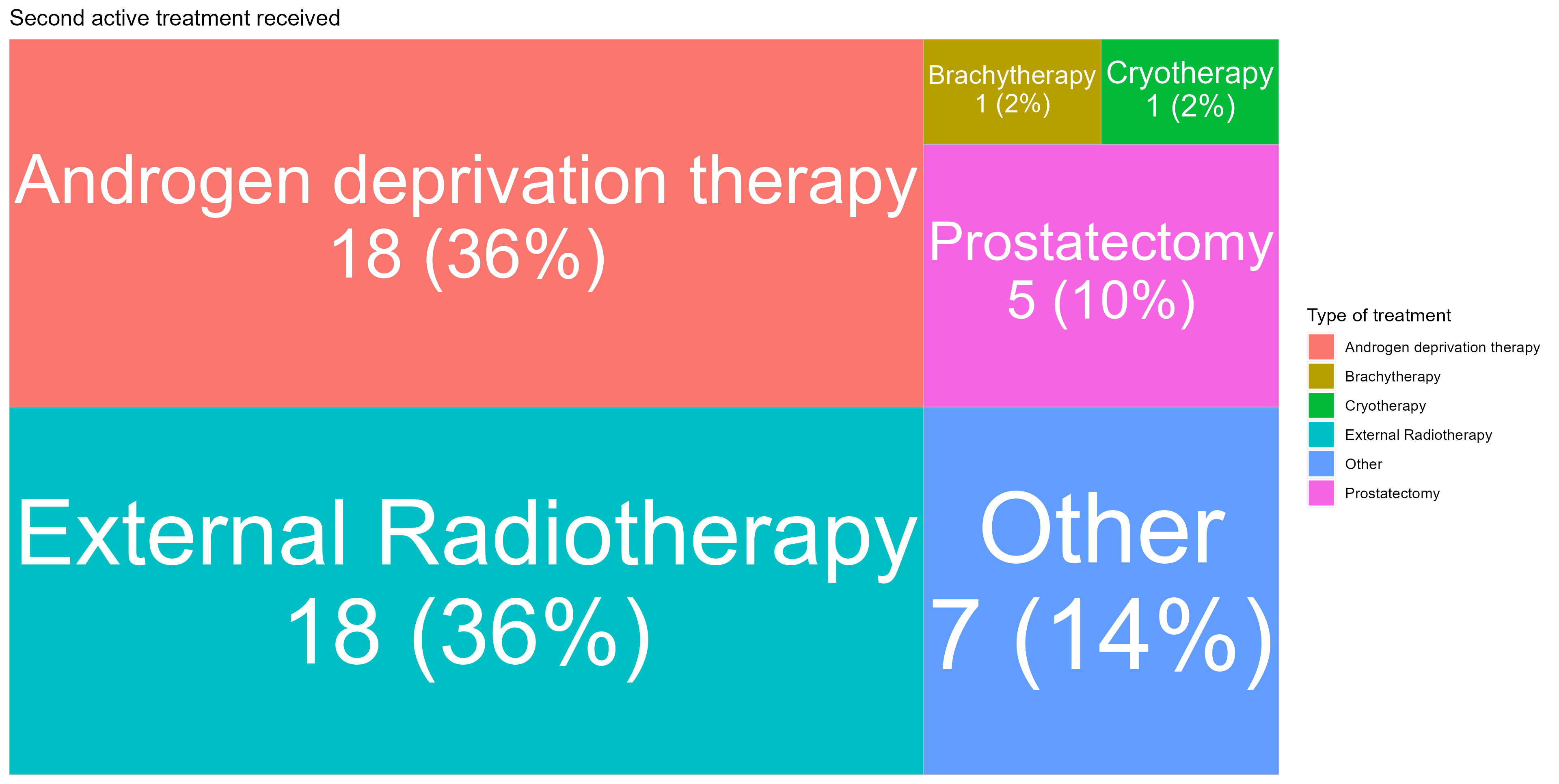

Supplement: Supplementary file 2 — Figure 1B. Second active treatment recieved. [file CAM4-14-e71173-s002.tiff]
